# Phenotypic, Metabolic and Genetic Adaptations of the *Ficus* Species to Abiotic Stress Response: A Comprehensive Review

**DOI:** 10.3390/ijms25179520

**Published:** 2024-09-01

**Authors:** Shengyun Yuan, Tianxiang Yin, Hourong He, Xinyi Liu, Xueyan Long, Pan Dong, Zhenglin Zhu

**Affiliations:** School of Life Sciences, Chongqing University, Chongqing 401331, China; 20211764@stu.cqu.edu.cn (S.Y.); 20211533@stu.cqu.edu.cn (T.Y.); 20211444@stu.cqu.edu.cn (H.H.); 202326021038t@stu.cqu.edu.cn (X.L.); 202326021039t@stu.cqu.edu.cn (X.L.)

**Keywords:** *Ficus*, abiotic stress, evolution, stress-resistance, mechanism

## Abstract

The *Ficus* genus, having radiated from the tropics and subtropics to the temperate zone worldwide, is the largest genus among woody plants, comprising over 800 species. Evolution of the *Ficus* species results in genetic diversity, global radiation and geographical differentiations, suggesting adaption to diverse environments and coping with stresses. Apart from familiar physiological changes, such as stomatal closure and alteration in plant hormone levels, the *Ficus* species exhibit a unique mechanism in response to abiotic stress, such as regulation of leaf temperature and retention of drought memory. The stress-resistance genes harbored by *Ficus* result in effective responses to abiotic stress. Understanding the stress-resistance mechanisms in *Ficus* provides insights into the genetic breeding toward stress-tolerant crop cultivars. Following upon these issues, we comprehensively reviewed recent progress concerning the *Ficus* genes and relevant mechanisms that play important roles in the abiotic stress responses. These highlight prospectively important application potentials of the stress-resistance genes in *Ficus*.

## 1. Introduction

**Figure 1:**
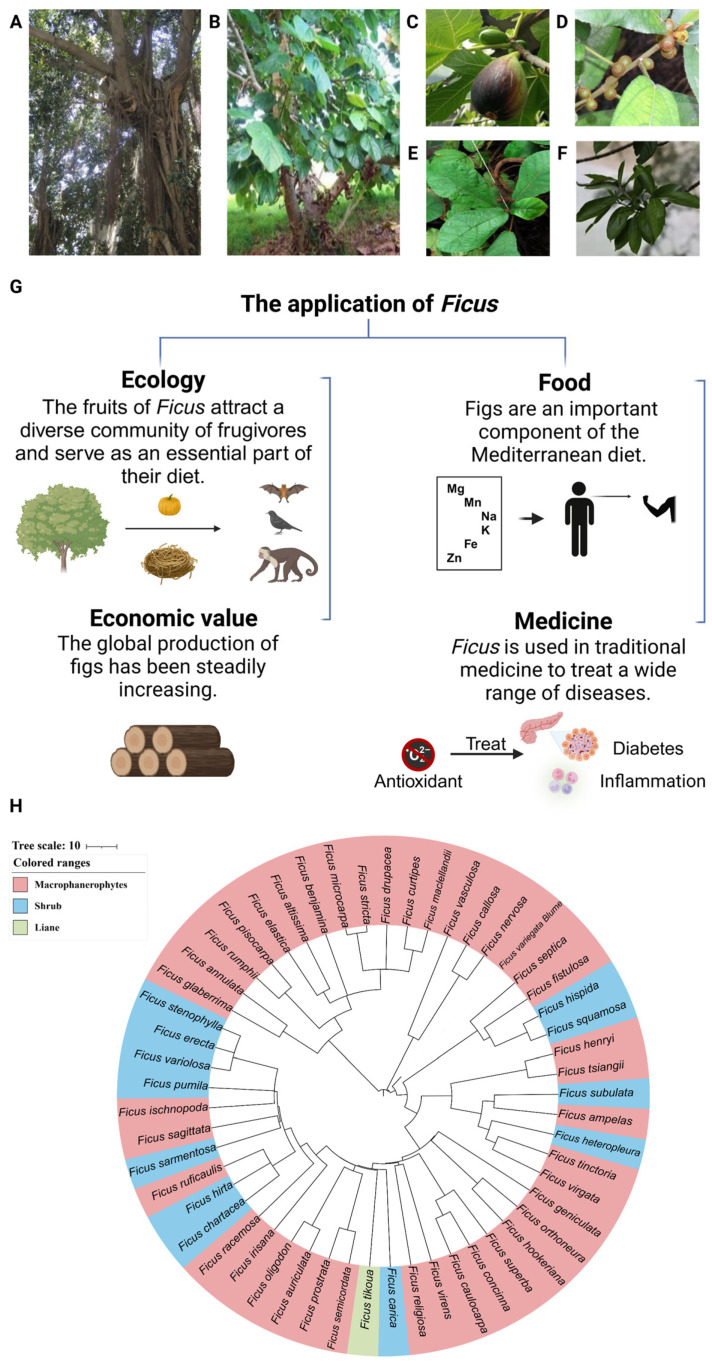
Morphology and application of the genus *Ficus*. (**A**) The aerial roots of *Ficus microcarpa* [23]. (**B**) The morphologies of *Ficus auriculata* [3]. (**C**) The fruits of *Ficus carica* [3]. (**D**) The fruits of *Ficus hirta* [3]. (**E**) The morphologies of leaves in *Ficus tikuoa* [3]. (**F**) The morphologies of leaves in *Ficus hispida* [4]. (**G**) The application of the *Ficus* species. (**H**) The species evolutionary tree of the *Ficus* species, based on documented taxonomy information in www.timetree.org (accessed on 13 June 2024).

The genus *Ficus* (Ficeae, Moraceae), commonly known as fig trees, is classified into six different subgenera: *Urostigma*, *Pharmacosycea*, *Ficus*, *Sycidium*, *Sycomorus*, and *Synoecia* [1]. The *Ficus* species originated from the tropics, extended into warm temperate zones [2], and displays interspecific morphological variations and adaptiveness to diversified environments. These result in the largest genera of woody plants [3,4,5] (Figure 1A–F). There are 880 documented species of *Ficus* to date [6]. The key characteristics of most *Ficus* trees include the secretion of white latex, a pair of stipules or a stipule sheath to protect the young branches, and a “tri-veined” shape, in which the basal lateral veins of the leaves exhibit an oblique orientation and form acute angles with the central vein.

The *Ficus* species have both ecological and economic importance [7,8] (Figure 1G). The *Ficus* trees play a crucial role in rainforest ecosystems [9]. *Ficus* spp. have been recognized as keystone plant resources that support diverse frugivorous vertebrate communities [10]. The fruits of *Ficus* spp. attract a diverse community of frugivores and provide a reliable diet for frugivore survival [11,12,13,14]. The fruits are crucial sustenance for fruit bats and primates, including capuchin monkeys, langurs, gibbons, and mangabeys, and are also vital for various bird species such as Asian barbets, pigeons, hornbills, fig-parrots, and bulbuls. The survival of these birds relies on the *Ficus* trees, resulting in the abundance of bird populations [12]. Figs are consumed in significant quantities by both avian and bat species [15]. Moreover, *Ficus* has become an important crop worldwide and brought enormous economic benefits [16]. The *Ficus* tree and its fruits also have food and medical uses [17] (Figure 1G). *Ficus carica*, the common fig, indigenous to the Mediterranean basin, bears fruits rich in nutrition [18,19]. This *Ficus* species is well-adapted to a variety of environmental conditions and holds significant economic value due to its fruits [8,20]. *Ficus deltoidea*, mistletoe fig, a shrub or small tree predominantly thriving in humid tropics and native to the Thai Peninsula and Malaysia, is rich in multiple antioxidant compounds and with medical applications in diabetes treatment, antioxidation, anti-inflammation, analgesics, and wound-healing promotion [21]. *Ficus deltoidea* is rich in essential elements, such as magnesium, manganese, potassium, sodium, iron, and zinc. The tea derived from this species supports the daily mineral requirements of the human body [22].

Other economically important fruits, such as apples, bananas, peaches, oranges, cherries, pears and coconut, are often severely impacted by environmental stress, but most *Ficus* species display superior resilience to stress [24,25,26,27,28,29,30]. The *Ficus* species are drought tolerant. They mostly thrive in sunlight and high temperatures, with an average annual temperature of >15 °C, growing degree days (GDDs) of 4800 °C, and an annual precipitation exceeding 1000 mm [31]. *Ficus insipida* tolerates extreme heat, with a thermal limit ranging from 50 to 53 °C [32]. Moreover, *F. carica* L. exhibits a salinity tolerance at a threshold of 100 to 150 mM NaCl [33]. The *Ficus* species can respond to drought stress by regulating leaf temperature [34] and increasing defoliation [35], and resist drought, flood and other stresses by forming adventitious roots [1,36,37,38]. Thus, understanding the mechanisms of *Ficus* responses to abiotic stress is crucial for breeding crop cultivars with enhanced stress resistance. The genetic resources of the *Ficus* species make potential contributions to the breeding of heat resistance cultivars in crops through genetic transformation [39,40,41]. These potentially assist in the application of the abiotic-stress-tolerant *Ficus* genes in genetically modifying economic crops in the future.

Due to the issues described above, it is important to review the advancements concerning the antioxidants and stress resistance of the *Ficus* species for further exploration and multi-field application of the stress resistance genes harbored by *Ficus*. However, there is a lack of relevant reviews to date. Consequently, we systematically summarize the recent advances in the research on the abiotic stress resistance of *Ficus* in terms of phenotypes, genetics and metabolism. We introduce the evolved abiotic stress tolerance of the *Ficus* species in terms of morphology, physiology and associated molecular mechanisms. These should contribute to further in-depth explorations of the gene pool of *Ficus*, such as identifying the potential genes with economic values and uncovering the abiotic stress regulatory network of plants, benefitting the application of the stress-resistance genes in crop breeding in the future.

## 2. Adaption of the *Ficus* Species to Tropical Environment

The evolutionary tree of *Ficus* species (Figure 1H), built on the basis of previous records [42], elucidates the evolutionary relationships of the *Ficus* species. Most *Ficus* species are diploid and possess 13 or 14 pairs of chromosomes (2*n* = 26, 2*n* = 28). The genomes of five *Ficus* species have been assembled to date (Table 1). The *Ficus* species have undergone extraordinary evolution in terms of plant life cycles. The *Ficus* species exhibit a diverse range of growth forms, from shrubby pioneers that reach a height of 1 to 2 m, to small and emergent trees, as well as climbers, epiphytes, and hemiepiphytes. They also include banyans with expansive canopies exceeding 150 m in diameter [43,44]. The lifespans range from a few years for pioneer shrubs to over a thousand years for larger trees [45]. The genomic diversity observed among different *Ficus* species indicates a high degree of environmental adaptability and genetic variability.

The adventitious roots of *Ficus* are crucial for respiration and adaptation to the high-rainfall, high-temperature, and high-humidity conditions characteristic of tropical environments. The main trunks and lateral branches of the *Ficus* trees frequently produce numerous adventitious roots that grow vertically downward. These roots may remain suspended or penetrate the soil upon reaching the ground, forming woody pillars that enhance the adaptation to tropical environments. Studies have shown that the formation and development of adventitious roots in the *Ficus* trees are associated with a light-induced molecular pathway that enhances auxin synthesis and transport capabilities [1].

The plant xylem serves three primary functions: mechanical support, water conduction, and storage of water and photosynthates [49]. The *Ficus* trees exhibit phenotypes distinct from those of other common crops. Fan et al. conducted allometric analysis on the leaves and stems of 28 *Ficus* species. The findings are as follows: (1) There is an isometric growth relationship between the size (area and weight) of twigs and leaves in *Ficus*, and an allometric growth relationship exists between petioles and leaves. (2) The intensity of the leaf production on twigs (the number of leaves supported per unit weight of twigs) has a negative isometric growth relationship with the leaf size. (3) There is a trade-off between the water conduction efficiency and the mechanical support capacity in *Ficus* twigs [50]. (4) The dual functions of the xylem in *Ficus* twigs, namely, water conduction and mechanical support, create a trade-off that determines the allometric growth pattern between the twig and the leaf size [50]. Compared to the deciduous *Ficus pisocarpa*, the evergreen *Ficus orthoneura* has smaller and denser vessels and stomatal diameter [51]. The thickness of the cuticle, upper epidermis, and hypodermis in the leaves of *Ficus orthoneura* is more than twice that of *Ficus pisocarpa*. The thickness of spongy tissue is over four times greater. The palisade tissue and lower epidermis show no significant difference in thickness. They both exhibit a high proportion of spongy tissue in their leaves. Compared with *Ficus pisocarpa*, *Ficus orthoneura* has a higher overall leaf thickness and a lower palisade tissue thickness to spongy tissue thickness (P/S) ratio [51].

Previous work identified 811 genes highly expressed in the tips of adventitious roots in the banyan tree through transcriptome analysis. These genes are significantly enriched in transport-relevant pathways [1]. Notably, several genes associated with auxin synthesis, regulation, and transport, such as PIN, GNOM, TAR, YUC, IAA14, ARF7/19, PLETHORA2, and WOX11, are highly expressed in adventitious roots [36]. Compared with other species lacking adventitious roots, such as *F. hispida*, mulberry, and Arabidopsis, several genes in *Ficus microcarpa* exhibit significant expansion. These genes function in auxin synthesis (YUC2, YUC6, and TAR), auxin signaling cascades (CLU1 and SINAT5), auxin transport (PIN1 and GNOM), and photoreceptors (CRY2 and PHR2). The gene GNOM is expressed at high levels in adventitious roots and plays a pivotal role in the endocytic recycling of the auxin efflux carrier protein PIN1. This suggests an exceptionally efficient transport mechanism for the apical auxin [52,53]. Metabolomic analyses reveal that adventitious roots of *Ficus microcarpa* accumulate a high level of IAA auxin (15.65 ng/g), five times higher than the auxin content found in the leaves of *Ficus microcarpa* and *F. hispida* [1]. Comparative genomic studies have identified extensive structural variations between the two species, such as chromosomal breakages and segmental duplications [1]. These results suggest that, under light stimulation, in a certain range, extensive auxin synthesis and transport occur in *Ficus microcarpa* and promote the formation and development of the adventitious roots. These variations are associated with critical biological processes, such as plant immunity and terpenoid synthesis, providing a genetic basis for adaptive evolution.

Here, we summarize the biological characteristics of *Ficus* in tropical environments, and focus on the formation of adventitious roots and the structures of branches and leaves. The *Ficus* species exhibit variations in branch and leaf structures, with complex xylem structures serving dual functions. There is a trade-off between the water conduction and the mechanical support capacity. The formation of adventitious roots is regulated by multiple genes, and a high expression of specific genes likely promotes the development. These are significant for understanding the adaptive evolution of *Ficus* in tropical ecosystems, and the interactions between *Ficus* and other organisms.

## 3. The Responses of *Ficus* to Abiotic Stress

Abiotic stress is the detrimental impact of non-living environmental factors on plants. The stress significantly impairs the growth, development, and metabolic processes, and thereby affects crop yields [54,55]. Abiotic factors account for more than 50% of the agricultural losses. With increases in the degree of urbanization and industrialization, arable land is progressively diminishing. Climate change could further reduce agricultural productivity by 10–25%, with losses being expected to increase over the next 50 years [56]. In this section, we describe the responses of several *Ficus* species to abiotic stresses, including high-temperature stress, drought stress, flood stress, oxidative stress, and salt stress. The corresponding types and specific mechanisms of *Ficus* species and stress involved in this part are shown in Table 2.

### 3.1. The Responses of Ficus to Heat Stress

In crop production, rising temperatures result in combined effects of drought and heat waves. These severely impact ecosystems and agricultural development [77]. High-temperature stress impacts plant growth at multiple developmental stages. During seed germination, high temperatures can hinder or completely halt the period, depending on the plant species and the severity of the stress. At the plant maturity stage, high temperatures can negatively affect photosynthesis, respiration, water balance, and cell membranes [78]. Additionally, high-temperature stress impacts hormone levels, as well as primary and secondary metabolism, potentially causing necrotic spots on leaves and stems, inhibiting growth and accelerating leaf senescence [79]. Heat stress also leads to the accumulation of ROS in chloroplasts, reduces antioxidant activity and potentially accelerates leaf abscission [80]. During growth and development, plants respond to heat stress by upregulating the expressions of various heat shock proteins, activating stress response proteins, and stimulating the production of ROS [81].

**Figure 2:**
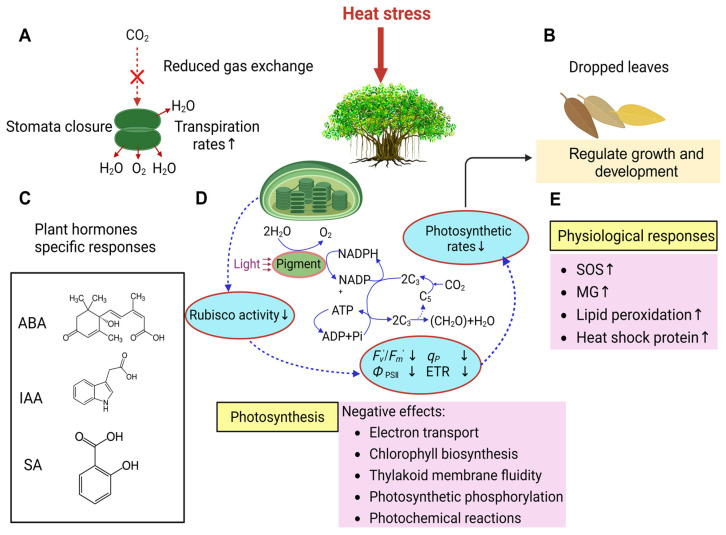
The responses of *Ficus* to heat temperature stress. (**A**) The decreases in stomatal conductance and the gas exchange rates, and the increases in the transpiration rate. (**B**) *Ficus* respond to high-temperature stress by dropping a large number of leaves. (**C**) Plant hormones including IAA, ABA, and SA are particularly responsive to high temperatures. (**D**) Heat stress impairs photosynthesis by negatively affecting electron transport, chlorophyll biosynthesis, thylakoid membrane fluidity, and photosynthetic, phosphorylation, and photochemical reactions. (**E**) Physiological responses to heat stress, including increased SOS and MG, increased levels of lipid peroxidation, and synthesis of heat shock protein.

Among the approximately 880 species in the genus *Ficus*, about 380 are classified as hemiepiphytes [82]. They begin the lifecycle as epiphytes and eventually establish aerial root connections with the ground [57]. Huayang et al. found that the hemiepiphytic *Ficus* exhibit greater tolerance to temperature and drought stress at the seedling stage, allowing them to secure resources more effectively in their ecological niches [57]. Under high-temperature conditions (35/25 °C), the hemiepiphytic *Ficus* trees show high germination and seedling survival rates. They enhance their germination peak slowly and have long germination periods, suggesting great drought tolerance in larger-sized categories of the hemiepiphytic *Ficus* trees [58]. Endogenous plant hormones, such as abscisic acid (ABA), indole-3-acetic acid (IAA), and salicylic acid (SA), exhibit specific responses to temperature stress at different regeneration stages in the hemiepiphytic *Ficus* trees (Figure 2C). For the non-hemiepiphytic *Ficus* species, the response of SA to temperature stress shows heightened sensitivity, as demonstrated by the significantly reduced SA levels during seed imbibition. No significant changes were observed during germination. Conversely, the increase in IAA in the *Ficus* seedlings exposed to high temperatures suggests that the temperature affects the synthesis of auxin or catabolism, thereby modulating growth responses [57].

*F. carica* L. responds to extreme heat stress by closing stomata and shedding leaves to avoid damages (Figure 2B). Under high temperatures and non-water-limited conditions, fig trees exhibit reduced photosynthetic rates (Figure 2A). Ammar et al. documented the seasonal variations in the physiological behavior of leaves in two fig cultivars, Zidi and Bither Abiadh [60]. The stomatal conductance (g_s_) of these two fig cultivars exhibits seasonal variation. Under high-temperature stress, figs reduce their gas exchange rates. In another study, the leaf temperature was identified as the primary factor limiting the gas exchange capacity of the fig trees growing under rain-fed conditions [61]. Statistical results of the seasonal variations in photosynthesis (P_n_) and transpiration rate (E) of the two fig cultivars suggest positive correlations across P_n_, E, and g_s_, consistently with previous findings on fig trees [62]. Indeed, temperature affects the physiological processes of figs, including photosynthesis and transpiration. Their interactions mitigate the adverse effects of abiotic stress on plant growth and development. Jin et al. investigated the effects of high-temperature treatments of different durations on *Ficus microcarpa* var. *microcarpa* in terms of gas exchange parameters, chlorophyll fluorescence parameters, and Rubisco activity [64]. The results indicate that prolonged high-temperature treatments result in continuous reductions in the net photosynthetic rate (P_n_) and slow decreases in stomatal conductance (g_s_). The transpiration rate (Tr) initially increases rapidly and then stabilizes, but the intercellular CO_2_ concentration (Ci) shows no significant change, suggesting that the decrease in P_n_ is not caused by stomatal factors. Further studies reveal that high-temperature stress leads a continuous decline in the efficiency of excitation energy capture by open photosystem II (PS II) reaction centers at moderate irradiance, photochemical quenching, the quantum yield of PS II electron transport and the electron transport rate (ETR). The initial Rubisco activity declines more rapidly than the fluorescence parameters. Non-photochemical quenching (NPQ) initially increases rapidly and then slightly decreases [64]. These results suggest that high temperatures inhibit photosynthesis in the *Ficus* trees primarily by suppressing Rubisco activity, which subsequently affects PS II activity and electron transport.

In addition to the impact on photosynthesis, high-temperature stress also affects several physiological indicators of the fig trees. Song et al. found that the treatment of the fig seedlings with 24-epibrassinolide (EBR) reduced the high-temperature-induced increases in ROS, methylglyoxal (MG), and lipid peroxidation levels. This study demonstrates that EBR mitigates heat-induced oxidative stress by enhancing non-enzymatic antioxidant responses, enzymatic antioxidant responses and the MG detoxification system (Figure 2E). The high temperature exerts damaging effects by inhibiting chlorophyll biosynthesis and inducing osmotic stress. The EBR pretreatment restores the effects, suggesting a protective role of EBR against heat damages [83].

High temperatures negatively affect the processes relevant to photosynthesis, including electron transport, chlorophyll biosynthesis, thylakoid membrane fluidity, photosynthetic phosphorylation, and photochemical reactions. Consequently, the damage caused by heat stress in chloroplasts results in the inactivation of heat-sensitive proteins, such as Rubisco activase, and the downregulation of essential chloroplast components, thereby reducing photosynthetic efficiency [82] (Figure 2D). The heat stress transcription factors (HSFs) play a critical role in regulating the expression of heat-responsive genes [84]. When plants are subjected to high-temperature stress, the acetylation lowers binding affinity (ALBA) proteins bind to a series of mRNAs, e.g., HSF mRNAs. ALBA proteins also recruit them into stress granules and processing bodies through phase separation. These protect HSF mRNAs from degradation under high-temperature conditions [85]. In the alba456 mutant, HSF mRNAs are not effectively recruited into stress granules and processing bodies for protection, resulting in the degradation by XRN4 from 5′ to 3′ in the cytoplasm and decreased heat tolerance in plants [86]. Genes encoding key transcription factors responsive to heat stress (HS), such as Heat Shock Factor A1 (HSFA1), HSFA2, Dehydration-Responsive Element-Binding Protein 2B (DREB2B), and Heat Shock Proteins (HSPs), are present in various plant species [87]. However, further research is needed to elucidate the specific mechanisms of high-temperature stress response in *Ficus*.

### 3.2. The Responses of Ficus to Drought Stress

Within the overwhelming trend of global climate change, reduced rainfall and increased evaporation rates are becoming universalized, and droughts are more common and severe than before in many regions [88,89]. Additionally, global warming has resulted in unpredictable rainfall patterns, causing prolonged drought periods to reemerge globally [90,91]. Drought inhibits cell division and root differentiation, alters plant morphology, and results in stunted growth, wilting, or reductions in leaves. Additionally, drought causes stomatal closure, which leads to reduced photosynthetic rates and the accumulation of ROS, resulting in irreversible cellular damages [92]. Stomatal closure is the initial response to drought stress and a protective mechanism against abiotic stress, preventing water loss through transpiration. This process is more closely related to soil water content than to leaf water status, and is mainly regulated by the chemical signals, particularly ABA, produced in dehydrated roots [93,94].

**Figure 3:**
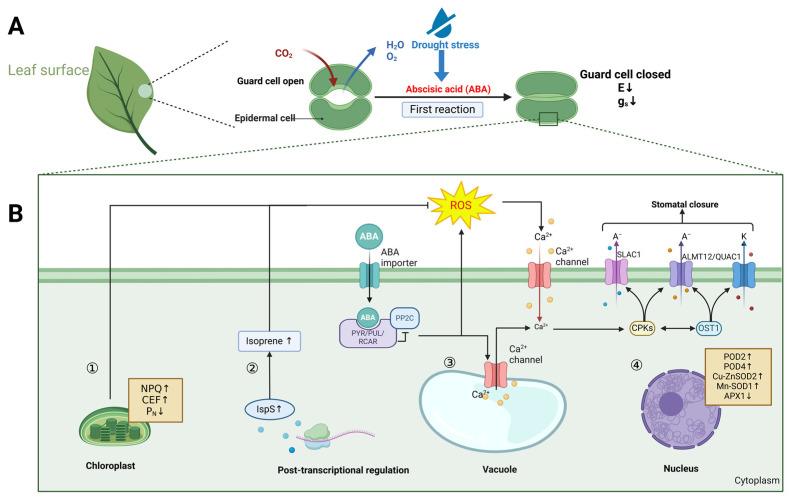
*Ficus* responses to drought stress. (**A**) The primary response of *Ficus* to drought stress was the closure of stomata by the endogenous plant hormone ABA. (**B**) The intracellular response consists of four parts: first, NPQ and CEF are enhanced, and the photosynthetic rate is decreased; second, the post-transcriptional regulation of IspS leads to an increase in the emission rate of isoprene; third, under the influence of drought stress, ABA is produced in leaves, which causes signal transduction and conformational changes in channel proteins at all levels; fourth, the transcription of POD2, POD4, Cu-ZnSOD2 and Mn-SOD1 increases, and the transcription of APX1 decreases. Together, the second and fourth steps enhance the scavenging capacity of ROS.

Compared with the non-hemiepiphytic *Ficus*, the hemiepiphytic *Ficus* exhibits greater drought tolerance. Hemiepiphytic *Ficus* employs more conservative water use strategies. Enhanced non-photochemical quenching (NPQ) and cyclic electron flow (CEF) have been recognized as crucial mechanisms to safeguard the photosynthetic apparatus during drought conditions. During peak drought periods, *Ficus racemosa* (a non-hemiepiphytic fig tree) shows significantly greater declines in the leaf turgor pressure, the maximum photosynthetic rate, and the maximum quantum yield of photosystem II compared to *F. tinctoria* (a hemiepiphytic fig tree). *F. racemosa* experiences net photoinhibition of PS II. Under seasonal drought conditions, enhanced NPQ and activated CEF contribute to the photoprotection of both PS I and PS II, especially in *F. racemosa* [65] (Figure 3B). Drought damages *Ficus* by disrupting thylakoid membranes, reducing the accumulation of dry matter and accumulating reactive oxygen. *Ficus* responds to drought stress by decreasing photosynthetic rate, reducing water loss, scavenging ROS, and defoliating.

The primary response of *F. carica* L. to drought stress is a reduction in stomatal conductance, resulting in controlling transpiration and photosynthetic rates, regulating the leaf temperature, and increasing the leaf drop to reduce water loss [35]. The primary response of *Ficus* to drought stress is the closure of stomata by the endogenous plant hormone ABA (Figure 3A). In the responses of *F. carica* L. to drought treatment, P_N_, g_s_, and E are decreased, along with reductions in the maximum quantum yield of photosystem II (*F_v_/F_m_*) and chlorophyll index. Drought stress causes an increase in leaf temperature and a decrease in leaf number in fig plants, while rewatering stimulates the emergence of new leaves and restores the photosynthetic function of previously stressed plants [35]. *F. carica* L. retains a memory of drought stress, allowing for rapid growth recovery after stress alleviation [60]. Abdolinejad et al. assessed the tolerance of the explants from two tetraploid fig cultivars, Sabz and Torsh, and the diploid control plants to water stress. Polyethylene glycol (PEG) was used to induce water stress [95]. Morphological, hormonal, physiological, and biochemical analyses indicate that both tetraploid genotypes exhibited superior water stress tolerance compared with the diploid control plants [95]. Early stomatal closure in fig trees helps to reduce transpiration and prevent hydraulic failure caused by xylem embolism [96]. However, prolonged water stress causes massive leaf drops in fig trees, along with a reduction in the percentage of persistent leaves by 10% [34]. Plants can survive by dropping leaves during drought stress, maintaining stem vitality and accumulating more carbohydrates in the stems and roots, which will stimulate the emergence of new leaves and regrowth when soil moisture levels return to normal. After rehydration following drought stress, fructose levels increase in leaves, while sucrose levels decrease in leaves and stems, indicating that the transformation from sucrose to fructose provides energy for bud regrowth [97]. The evidence indicates that the employment of ploidy manipulation techniques in the breeding of figs may be an effective strategy to develop novel and desirable traits endowing enhanced tolerance to abiotic stresses, thus addressing the limitations of traditional breeding.

Drought affects the physiological metabolism and photosynthesis of *Ficus*. *Ficus deltoidea* is susceptible to drought stress, leading to leaf damage. The foliar application of hydrogen peroxide can mitigate the negative effects of drought stress, alleviating it through improving the chlorophyll content, transpiration rate, and stomatal conductance [66]. *Ficus septica* exhibits increased isoprene emission rates under short-term drought stress, with a decrease in photosynthetic rates by 90.6%, a decrease in stomatal conductance by 99.5%, and a decrease in transpiration rates by 82.3% [67]. In *Ficus concinna*, the cyclic electron flow around PS I plays a critical role in generating the pH gradient (ΔpH) across the thylakoid membrane, effectively dissipating excess excitation energy under high-temperature conditions. The cyclic electron transport around PS I is a crucial photoprotective mechanism that allows the photosynthetic apparatus to adapt to high temperatures [71]. *Ficus benjamina* L. experiences thylakoid structure disruption in its leaves and reduced dry matter accumulation after drought stress [68].

*Ficus* primarily responds to drought by reducing water loss. The evergreen species *Ficus virens* exhibits conservative water use and drought-tolerant strategies: its branches exhibit low xylem hydraulic conductivity and strong embolism resistance, ensuring hydraulic safety. During drought, it reduces leaf water loss and compensates for reduced photosynthetic capacity by sustaining carbon fixation in the leaves. In contrast, the deciduous species *F. racemosa* adopts risky water use and drought-avoidance strategies, maintains leaf turgor through elastic adjustment, and avoids water loss and xylem cavitation by shedding leaves [51].

In addition, the *Ficus* species responds to drought stress by enhancing its antioxidant resistance. Under drought stress, *Ficus septica* exhibits the increased transcription of antioxidant genes such as peroxidase 2 (POD2), POD4, copper-zinc superoxide dismutase 2 (Cu-ZnSOD2), and manganese superoxide dismutase 1 (Mn-SOD1). The transcription of ascorbate peroxidase 1 (APX1) decreases (Figure 3B). The regulation of isoprene biosynthesis in *F. septica* under drought stress is not directly associated with the antioxidant defense network. The post-transcriptional regulation of isoprene synthase (IspS) leads to changes in isoprene emission rates (Figure 3B), enhancing the scavenging capacity of ROS. Combined with increased antioxidant enzyme activity, the changes contribute to the drought stress response in *F. septica* [67]. Heat treatments at 80 °C eliminate the activity of dehydroascorbate reductase (DHAR) in *Ficus microcarpa* L. f. cv. Golden Leaves [51].

Plant responses to drought stress involve protective mechanisms, such as stomatal closure and leaf abscission. Different plant species employ various water-use strategies to cope with drought. Additionally, ploidy manipulation techniques can develop new drought-tolerance traits, offering new avenues for plant breeding. These are expected to provide important theoretical foundations and practical guidance for addressing the survival and growth of plants in drought environments.

### 3.3. The Responses of Ficus to Flood Stress

Flooding, as an abiotic stress, affects the composition and productivity of numerous plant communities worldwide [98]. The Group VII Ethylene Response Factors (ERF-VIIs) constitute a category of ERF transcription factors (TFs) and modulate the expression of numerous genes associated with adaptive responses to flooding and hypoxia [72]. During the periods of flood stress, respiration is compromised, resulting in the generation of ROS. In order to preserve the balance of ROS, the submerged plants activate oxidoreductase enzymes, including plastocyanin [99]. Transcriptomic studies consistently reveal that submergence stress induces an upregulation of gene clusters associated with the responses of plants to fungi and bacteria. These expressed genes encode the pattern recognition receptors (PRRs), wall-associated kinases (WAKs), leucine-rich repeats (LRRs), and lectin-DUF26 proteins [100,101,102].

*Ficus* has high vegetation coverage in the seasonally flooded areas of the Pongolo River Floodplain, indicating a great flood tolerance [103]. Similarly, in the upper reaches of the Peruvian Amazon River, *Ficus* adapts to seasonal flood stress by forming aerial prop roots, as previously mentioned [72]. However, *Ficus tikoua* does not exhibit strong water tolerance. Survival rate analyses indicate that the winter tolerance of these plants is affected by the duration and depth of flooding [73]. Research has indicated that the distribution of *F. squamosa* is predominantly near streams. This species is a rheophytic shrub characterized by rooting stolon-like stems, especially found in fast-flowing streams. Field observations suggest that this species can endure significant disturbances during flood seasons. Additionally, *F. squamosa* shows anatomical adaptations that facilitate seed dispersal via water; its seeds can float and disperse over considerable distances, reflecting adaptation to flood conditions [74,75].

Flooding poses a severe threat to plant survival by hindering various physiological and metabolic activities in *Ficus* and disrupting normal growth and development. These disruptions include reduced stomatal conductance, CO_2_ assimilation rate, photosynthesis rate, and nutrient imbalance, leading to decreased crop yields. *Ficus* exhibits various morphological, anatomical, and physiological adaptive traits under submergence conditions, such as forming adventitious roots, enhancing ethylene production, and increasing alcohol dehydrogenase (ADH) activity and proline content [37]. These indicate that *Ficus* indeed has superior flood tolerance compared with other plants. However, under prolonged flood stress, plants exhibit oxidative damages due to excessive ROS production. ROS interferes with normal metabolism and reduces membrane integrity by oxidizing proteins, nucleic acids, and DNA [104]. In such cases, *Ficus* develops an antioxidant defense system, including enzymatic and non-enzymatic antioxidants, to scavenge excess ROS. However, there is a lack of comprehensive reports on flood response mechanisms in *Ficus*.

### 3.4. The Responses of Ficus to Oxidative Stress

A primary indicator of abiotic stress at the molecular level is the increased production of reactive oxygen species (ROS), including singlet oxygen (^1^O_2_), superoxide (O_2_^•−^), hydrogen peroxide (H_2_O_2_), and hydroxyl radicals (OH^•^). Low levels of ROS function as signaling molecules and promote plant tolerance to abiotic stress by regulating the expression of defense-related genes [105]. ROS are primarily generated in the rapidly developing regions of adventitious roots, where NADPH oxidase, POD, and SOD play crucial roles in regulating the production of ROS and the development of roots. The activities of POD and the hydroxyl radical cycle are particularly significant in the production of ROS within adventitious roots [38]. Under AH, the production of H_2_O_2_ in *Ficus religiosa* L. increases by 55%, and the activity of POX increases by approximately 30%. The activity of cytoplasmic POX increases by 11% compared with that of the cell-wall-bound POX. The activity of CAT increases approximately 2-fold during the day. The deposition of H_2_O_2_ in *F. religiosa* is higher at night than during the day, associated with increased activity of the nocturnal CAT. The decline in the aerial root cell viability is primarily due to decreased cellular pH, mineral assimilation imbalance, and the damage to cell walls, plasma membranes and signaling pathways [76]. Heavy metal pollution induces oxidative stress and cell death in adventitious roots of fig trees. Regarding oxidative damage, O_2_^•−^ is the initial and most important ROS in the root meristem, cortex, and even medullary cells [106]. *F. microcarpa*’s adventitious roots are sensitive to pollutant-induced ROS, with an accumulation of O_2_^•−^ in the roots under pollutant exposure. The cytochemical localization further confirms that the generated O_2_^•−^ is primarily present in the root cortex and medullary cells, resulting in increased lipid peroxidation and malondialdehyde [70].

### 3.5. The Responses of Ficus to Saline-Alkali Stress

Soil salinization, a severe consequence of climate change, is a global issue of concern [107,108,109,110], though there are significant differences across countries [111]. For poorly adapted plants, the prolonged submergence of tissues causes oxygen deficiency because oxygen diffusion in the water is much slower than in the air [112]. Additionally, excess water alters the physical and chemical properties of the soil, changes the types of soil microorganisms, and significantly affects the plant physiological metabolism and hormone levels in plants [98]. The pathways of salt-induced stress can be categorized into three types: osmotic, ionic, and secondary. Osmotic stress is caused by the high concentration of salts in the soil and water. Excess soluble salts in the soil lower the water potential at the root surface, reduce water uptake of the plant and result in water deficiency. Ionic stress results from the toxic effects of salt ions within plant cells. Salts absorbed by the roots are transported long distances through the transpiration stream to the shoots and eventually accumulate in the leaves. High concentrations of Na^+^ in the cytoplasm disrupt the uptake of other ions in plant cells, adversely affecting many metabolic pathways. Potassium (K^+^) is essential for the catalytic activity of many enzymes. Osmotic and ionic stress can lead to secondary stress in plants, including accumulating toxic compounds and disrupting the nutrient balance [113].

Under moderate salt stress (100 mM NaCl), the transcription levels of the genes involved in the transport of soluble carbohydrates generally increase in *F. carica* L., resulting in elevated levels of sucrose and d-sorbitol (an isomer of mannitol) in *F. carica* L. This affects the flavor of the fruit. Additionally, d-sorbitol acts as an osmolyte to regulate salt and drought stress. Moreover, previous studies have shown that the enhanced transcription of the mannitol-encoding gene OeMaT1 causes the accumulation of mannitol, which plays multiple roles in oxidative stress resistance and salt tolerance [18]. Elevated levels of saltadversely affect the root biomass, resulting in a reduction in the content of the chlorophyll. The fig cultivar Khdari shows a notable decrease of approximately 26% [33]. Additionally, the salt stress is associated with a sharp decline in the stomatal conductance and the net photosynthetic rate in common fig.

Under salt stress, we see an upregulated expression of the ROS signaling proteins, the receptor-like proteins, proline synthesis-encoding genes, and various transporter and channel-encoding genes in *F. carica* L. [63]. Additionally, cell wall components change in response to salt stress. Metabolically, salt stress causes an overall downregulation, including the decreased expression of genes involved in glycolysis, downregulated lipid metabolism, and increased amino acid metabolism. These indicate that *F. carica* L. exhibits moderate salt tolerance, with a safe growth range of 0 to 100 mM NaCl [46,63]. The salt-regulated genes include the novel genes previously not seen as associated with salinity response [33]. Within 24 days of salt stress, the genes encoding ROS signaling-related proteins, such as the tubby-like protein 8 and the nodulation signaling pathway 1, were upregulated, and the receptor-like protein cytosolic serine/threonine-protein kinase RBK2 was overexpressed. After 48 days of salt stress, the genes encoding delta-1-pyrroline-5-carboxylate synthase (P5CS), involved in proline biosynthesis and abiotic stress resistance, and the genes encoding raffinose synthase family proteins were overexpressed. The genes encoding transporters (e.g., bidirectional sugar transporter SWEET16, sugar transporter ERD6-like 6, transmembrane amino acid transporter) and channels (S-type anion channel SLAH3) were overexpressed, and are possibly involved in the regulation of salt stress [63]. The gene FcSPPB2 was expressed to a degree in Khdari and Zraki, but was overexpressed in Mwazi, reaching about 128-fold under excessive salt stress. The FcDREB gene was overexpressed in Khdari and Zraki after salt stress. Salt stress downregulated FcCIPK11 in Khdari but slightly upregulated Mwazi in Zraki. The expression of sorbitol dehydrogenase (FcSORD) shows a 246-fold increase in Zraki after salt stress. The gene FcDHN has a similar overexpression trend in the Zraki local cultivar. Mwazi and Zraki display remarkable overexpressions of a distinct array of salinity-responsive genes, highlighting the genetic diversity within the *Ficus* genus in response to salt stress [33]. Above all, we summarize the mechanisms of the *Ficus* response to abiotic stresses in Table 3.

## 4. The Adaptive Genes and Molecular Mechanism of *Ficus* Relating to Abiotic Stress

The proteins associated with plant abiotic stress response have been preliminarily identified in *Ficus*. The proteins include Glutathione S-transferases (GSTs), Papain-like cysteine proteases (PLCPs), and basic helix–loop–helix (bHLH). However, the specific function and mechanism of these proteins remains unknown. GSTs are associated with abiotic stress and regulate the plants in encountering cold, salt, and drought stresses [114]. Longbo Liu et al. identified 53 *GST* gene members in the fig (*F. carica* L.) genome. They found that the promoter regions of the *FcGST* gene family members contain at least one stress-responsive element, which may be associated with anaerobic stress, drought, and cold. However, the molecular mechanisms underlying their positive role in enhancing abiotic stress tolerance are still unclear. PLCPs are the most abundant cysteine protease family in plants, and play essential roles in abiotic stress responses. Here, 31 PLCP genes, designated as FcPLCPs, have been identified within the genome of the fig (*Ficus carica* L.). These FcPLCPs vary in length (from 1128 to 5075 bp) and contain 1 to 10 introns. Their coding sequences range from 624 to 1518 bp, encoding the proteins with a length of 207 to 505 amino acids. Subcellular localization analyses have shown that 24, 2, and 5 PLCP proteins are targeted to the lysosome/vacuole, cytoplasm, and extracellular matrix, respectively. Analyses of the promoters (upstream 2000 bp) of FcPLCPs have revealed numerous plant hormones and cold-responsive elements [115]. The bHLH transcription factor family, the second largest in plants, plays a pivotal role in a multitude of growth and developmental processes within these organisms. A total of 118 bHLH genes were identified from fig (*F. carica* L.) by a whole-genome database search [116]. Miaoyu Song et al. predicted at least 16 cis-regulatory elements in the promoter region of FcbHLH genes in fig (*F. carica* L.), which are involved in the response to various abiotic stresses (light deprivation, drought, low temperature, anaerobic conditions, defense, and stress). They identified light-responsive and anaerobic-induced abiotic stress regulatory elements and MYB binding sites in the promoters of five bHLH genes (FcbHLH8, FcbHLH24, FcbHLH83, FcbHLH46, and FcbHLH21) [116]. Kim et al. identified two major proteins closely associated with catalytic active rubber particles, peroxidase (POX) and trypsin inhibitor (TRI). They isolated a cDNA encoding a basic class I chitinase (CHI) from the latex of fig trees. Wounding strongly induced the expression of these three stress-related genes. Previous findings show that drought induces the overexpression of POX and downregulates the expression of CHI and TRI. Cold treatment slightly reduces the transcription levels of these genes, while salt stress slightly decreases the expression of CHI [117].

Some pathways that may be present in *Ficus* in response to abiotic stresses have not been reported. Kinases play a crucial role in abiotic stress signal transduction [118]. Moreover, other post-translational factors are becoming essential, and rapid mechanisms for regulating abiotic stress responses and the ubiquitin-mediated proteolytic system play a role in drought stress tolerance [119]. Root structure and growth affect plant tolerance to various stresses, particularly drought and nutrient deficiency [119]. However, there is a lack of research on the relevant kinases or post-translational factors involved in abiotic stress in *Ficus*. In the future, in-depth research on these abiotic stress response genes will further elucidate the specific mechanisms, allowing us to perform targeted modifications or artificially select superior genes. It is possible to develop crops with enhanced resistance to natural disasters using transgenic technology

## 5. Potential Application of *Ficus* Genes in Genetic Breeding

Understanding the molecular mechanisms of the genus *Ficus* in response to abiotic stress has profound implications in enhancing crop resilience. The exceptional stress resistance characteristics of *Ficus* offer significant insights for improving crop adaptation to adverse environmental conditions and ensuring yield stability. We suggest that incorporating salt-tolerance genes from *Ficus* into crops can enhance the adaptiveness to saline soils and increase yields. With the use of gene editing technologies, such as CRISPR/Cas9, it is possible to directly edit the genome of target crops by inserting or modifying stress resistance genes according to the stress resistance genes of *Ficus*, thereby conferring similar resilience to the target crops. Alternatively, plant tissue culture and transgenic techniques can be utilized to introduce stress resistance genes from *Ficus* into target crops through genetic engineering, thus imparting the desired resilience. The use of modern biotechnological methods to conduct comprehensive genomic research on *Ficus* can prospectively uncover more stress resistance genes, providing candidate genes for crop breeding. Future comprehensive studies of *Ficus* using genomics, transcriptomics, proteomics, metabolomics, and systems biology will be crucial for advancing crop resilience and broaden plant stress resistance research.

## 6. Conclusions

In this review, we systematically summarize recent studies on abiotic stresses in *Ficus*, providing a comprehensive and integrated perspective for future research on stress resistance in this genus. Current research on the abiotic stress resistance of *Ficus* focuses on morphological traits, physiological responses, biochemical processes, and genomic-level adaptations. *Ficus* trees exhibit local adaptive evolution characteristics and high genetic diversity. When faced with various stresses, *Ficus* responds through morphological evolution and physiological/biochemical pathways [37,42,60,66,98]. Under high-temperature and drought stress, *Ficus* exhibits physiological responses, such as reduced stomatal conductance, decreased net photosynthetic rate, accumulation of ROS, increased isoprene emission, and behavioral responses, such as increased leaf temperature and leaf drop. *Ficus* mitigates the negative impacts of flooding stress, such as reduced photosynthetic rates and ROS accumulation, by producing adventitious roots and increasing ethylene and proline content [67,80,95]. In response to oxidative stress, the *Ficus* trees increase the content of H_2_O_2_ and the activities of POX and CAT [76]. In response to salt stress, *Ficus* enhances the expression of osmolytes, upregulates amino acid metabolism, and downregulates lipid metabolism [46,63]. With advances in genomics, the reference genomes of five *Ficus* species have been assembled [1,42,46,47,48]. Based on the genomes, the genes and pathways potentially associated with resistance in the *Ficus* species were identified [33,114,115,116]. However, the specific regulatory networks remain unknown. There are several proteins and signal pathways that are relatively common in the response process of plants to adversity, which potentially function importantly in stress resistance networks. The identified stress-resistance genes and transcription factors in *Ficus* provide options to develop transgenic crops with enhanced stress-resistance capability [114,115,116]. The investigation of the abiotic stress response mechanisms should enhance the understanding of the stress resistance mechanisms in plants, and provide new insights for breeding resilient crop varieties [24,25,26,27,28,29,30].

The *Ficus* species provide research potentials. However, current relevant studies have limitations and face challenges. Previous research on *Ficus* generally focused more on economic value and medical applications. The mechanisms and specific pathways involved in stress tolerance require further exploration. The *Ficus* occupies a wide range of ecological niches, but their unique morphology (such as aerial roots) and large size pose challenges in studying the stress resistance, such as the application and control of adverse conditions, the measurement methods for physiological indicators, long research cycles, and the difficulty in replicating experimental conditions [1,36,37,38,69,70]. These require innovative approaches and advancements in technology and instrumentation to be addressed in the future.

## Figures and Tables

**Table 1:** The genome list of five *Ficus* species.

Latin Name	Level	GenBank	Release Date	Genome Size	References
*Ficus carica*	Chromosome	GCA_009761775.1	December 2019	333.4 Mb	[46]
*Ficus microcarpa*	Chromosome	GCA_025413485.1	September 2022	426.6 Mb	[1]
*Ficus hispida*	Chromosome	GCA_025413025.1	September 2022	369.8 Mb	[1]
*Ficus religiosa*	Scaffold	GCA_024759925.1	August 2022	406.1 Mb	[47]
*Ficus erecta*	Contig	GCA_008635985.1	September 2019	595.8 Mb	[48]

**Table 2:** The *Ficus* species and associated abiotic stresses referred to in this review.

Species	Response	Stress	References
*Hemiepiphytic Ficus*	Morphology, behavior, physiology	Heat, drought	[57,58,59]
*Ficus carica* L.	Morphology, behavior, physiology	Heat, drought, salt	[18,33,60,61,62,63]
*Ficus chartacea* var. *torulosa*	Physiology	Heat	[64]
*Ficus tinctoria*	Physiology	Drought	[65]
*Ficus racemosa*	Physiology	Drought	[65]
*Ficus deltoidea*	Physiology	Drought	[66]
*Ficus septica*	Physiology	Drought	[67]
*Ficus benjamina* L.	Physiology, cytology	Drought	[68]
*Ficus orthoneura*	Morphology	Drought	[51]
*Ficus microcarpa*	Physiology, morphology	Drought, oxidative	[69,70]
*Ficus concinna*	Molecular mechanisms	Drought	[71]
*Ficus* ssp.	Morphology	Flood	[72]
*Ficus tikoua*	Viability	Flood	[73]
*Ficus crytophylla*	Behavior	Flood	[74]
*Ficus squamosa*	Behavior	Flood	[75]
*Ficus religiosa* L.	Physiology	oxidative	[76]
*Hemiepiphytic Ficus*	Morphology, behavior, physiology	Heat, drought	[57,58,59]

**Table 3:** Effects of abiotic stress on *Ficus* and the responses.

Stress	Responses	
Heat stress	Morphology	Stomatal closure and leaf abscission.
	Physiology	Decreased photosystem activity; reduced photosynthetic rate; increased transpiration rate; elevated levels of IAA, ROS, MG, and lipid peroxidation.
	Cytology	Reduced chlorophyll synthesis.
	Molecular	Inactivation of heat-sensitive proteins; synthesis of heat shock proteins.
Drought stress	Morphology	Regulated leaf temperature, increased leaf abscission, reduced stomatal conductance; decreased root hydraulic conductance (Lp); and unchanged leaf turgor pressure.
	Physiology	Decreased photosynthetic and transpiration rates; accumulation of dry matter; reduced glutamine; enhanced non-photochemical quenching (NPQ); activation of cyclic electron flow (CEF) and increased isoprene emission rate.
	Cytology	Reduced chlorophyll synthesis and damaged thylakoid structure.
	Molecular	Increased transcription of POD2, POD4, Cn-ZnSOD2, and Mn-SOD1; decreased transcription of APX1.
Flood stress	Morphology	Seed dispersal via water; reduced stomatal conductance; formation of aerial prop roots.
	Physiology	Nutrient imbalance; accumulation of ROS; decreased photosynthetic rate; increased ethylene production.
	Cytology	Damaged membrane integrity.
	Molecular	Increased ADH activity and proline content.
Oxidative stress	Morphology	Decreased antioxidant capacity during senescence.
	Physiology	Increased hydrogen peroxide and malondialdehyde levels, increased POX activity; and lipid peroxidation.
	Cytology	Decreased cell viability in adventitious roots; damage to the cell wall and plasma membrane.
	Molecular	Ascorbate-glutathione (AsA-GSH) pathway.
Salt stress	Morphology	Reduced stomatal conductance.
	Physiology	Decreased photosynthetic rate; increased sucrose and d-sorbitol; downregulated glycolytic metabolism.
	Cytology	Decreased chlorophyll content; altered cell wall composition.
	Molecular	Increased transcription of carbohydrate transport genes; overexpression of ROS signaling proteins and proline synthesis coding genes.
